# Transcriptome characterization and high throughput SSRs and SNPs discovery in *Cucurbita pepo *(Cucurbitaceae)

**DOI:** 10.1186/1471-2164-12-104

**Published:** 2011-02-10

**Authors:** José Blanca, Joaquín Cañizares, Cristina Roig, Pello Ziarsolo, Fernando Nuez, Belén Picó

**Affiliations:** 1 Institute for the Conservation and Breeding of Agricultural Biodiversity, Universidad Politécnica de Valencia (COMAV-UPV), Camino de Vera s/n, 46022 Valencia, Spain

## Abstract

**Background:**

*Cucurbita pepo *belongs to the Cucurbitaceae family. The "Zucchini" types rank among the highest-valued vegetables worldwide, and other *C. pepo *and related *Cucurbita spp.*, are food staples and rich sources of fat and vitamins. A broad range of genomic tools are today available for other cucurbits that have become models for the study of different metabolic processes. However, these tools are still lacking in the *Cucurbita *genus, thus limiting gene discovery and the process of breeding.

**Results:**

We report the generation of a total of 512,751 *C. pepo *EST sequences, using 454 GS FLX Titanium technology. ESTs were obtained from normalized cDNA libraries (root, leaves, and flower tissue) prepared using two varieties with contrasting phenotypes for plant, flowering and fruit traits, representing the two *C. pepo *subspecies: subsp. *pepo *cv. Zucchini and subsp. *ovifera *cv Scallop. *De novo *assembling was performed to generate a collection of 49,610 *Cucurbita *unigenes (average length of 626 bp) that represent the first transcriptome of the species. Over 60% of the unigenes were functionally annotated and assigned to one or more Gene Ontology terms. The distributions of *Cucurbita *unigenes followed similar tendencies than that reported for *Arabidopsis *or melon, suggesting that the dataset may represent the whole *Cucurbita *transcriptome. About 34% unigenes were detected to have known orthologs of *Arabidopsis *or melon, including genes potentially involved in disease resistance, flowering and fruit quality. Furthermore, a set of 1,882 unigenes with SSR motifs and 9,043 high confidence SNPs between Zucchini and Scallop were identified, of which 3,538 SNPs met criteria for use with high throughput genotyping platforms, and 144 could be detected as CAPS. A set of markers were validated, being 80% of them polymorphic in a set of variable *C. pepo *and *C. moschata *accessions.

**Conclusion:**

We present the first broad survey of gene sequences and allelic variation in *C. pepo*, where limited prior genomic information existed. The transcriptome provides an invaluable new tool for biological research. The developed molecular markers are the basis for future genetic linkage and quantitative trait *loci *analysis, and will be essential to speed up the process of breeding new and better adapted squash varieties.

## Background

The botanical family Cucurbitaceae, commonly known as cucurbits, includes several economically and nutritionally important vegetable crops cultivated worldwide, such as cucumber, melon, watermelon and pumpkins, gourds and squashes [1]. The cucurbit family displays a rich diversity of many traits, being primary models for sex expression analysis, for the study of vascular biology and for the analysis of the mechanisms involved in fruit ripening [2-5].

Despite the agricultural and biological importance of cucurbits, knowledge of their genetics and genome has been very limited till now. So far, genomic efforts have largely focused on cucumber and melon. Recently, the whole genome sequencing of the cucumber, *C. sativus *var. *sativus *L., has been completed by combining traditional Sanger and next-generation Illumina GA sequencing technologies [6]. Also an effort is in progress through a Spanish Initiative to obtain the whole genome sequence of melon, *Cucumis melo *L. [7]. Many genomic resources are available for both crops and also for watermelon, *Citrullus lanatus *(Thunb.) Matsum. & Nakai. BAC libraries, collections of genetic markers, detailed physical and genetic maps, mapping populations, microarrays, sequence databases and mutant collections [8-11] are facilitating the use of cucurbits by the research community. Many genomic resources are available at the web site of the International Cucurbit Genomics Initiative (ICuGI) [12].

*Cucurbita *genus (2n = 2 × = 40), that include squashes, gourds and pumpkins, has been less studied. It contains some of the earliest domesticated plant species [13]. Today, three of them, *C. pepo *L., *C. moschata *Duchesne, and *C. maxima *Duchesne, have considerable impact on human nutrition, being appreciated by their nutritional and medical properties [14-17]. In addition to the use of the edible fruits, flowers, leaves, and vine tips are consumed, and seeds are also important as snacks, as a source of edible oil and protein for human and animal consumption, and in the pharmaceutical industry. Squashes are also popular as containers and for ornamental purposes. The economic value of *Cucurbita spp*. as rootstocks for overcoming soil borne diseases in cucurbits is significant [18].

*C. pepo *is the most economically important species within the genus distributed worldwide, and one of the most variable in the plant kingdom. Cultivated *C. pepo *is considered to comprise two subspecies each one including several cultivar-groups, ssp. *pepo *(Pumpkin, Vegetable Marrow, Cocozelle, and Zucchini) and ssp. *ovifera *(Acorn, Scallop, Crookneck, and Straightneck) [19,20]. Its great economic value is based mainly on the culinary use of the immature fruits as vegetables, often referred to collectively as "summer squashes", but also the Pumpkin and Acorn groups display a major use as mature fruits, known as "winter squashes". The great diversity of uses makes breeding objectives quite variable.

The currently available genetic and genomic tools for *Cucurbita *are very limited. Until now three genetic maps have been constructed: two maps from inter-specific crosses between *C. pepo *and *C. moschata *[21,22] and the third from an intra-specific cross of *C. pepo *(a USA oil-Pumpkin variety and an Italian Crookneck variety) [23]. These maps contained mostly RAPDs (Random Amplified Polymorphic DNA) and AFLPs (Amplified Fragment Length Polymorphism) markers. Only recently a collection of genomic microsatellites (Simple Sequence repeats, SSRs) has been developed and used to increase the map density [24]. The last map version comprises 178 SSRs, 244 AFLPs, 230 RAPDs, and two morphological traits (*h *(*hull-less *seed) and *Bu *(*Bush growth habit*). It contains 20 linkage groups with a map density of 2.9 cM and genome coverage of 86.8%. These SSRs were also used to study synteny between *C. pepo *and *C. moschata *[25].

The lack of denser genetic maps, larger high-throughput marker collections, and suited mapping populations is limiting gene isolation and squash breeding. Many *C. pepo *genes have been reported, mainly related to fruit quality and resistance to poty- and other viruses and several fungi, such as downy and powdery mildew [26], but only the transcripts of a few have been cloned and molecularly characterized in individual studies in *C. pepo *or other *Cucurbita *spp, for example genes involved in the biosynthesis or signaling pathways of growth regulators, affecting plant development, sex expression and response to stress [27-32].

Single nucleotide polymorphisms (SNPs) are the most abundant variations in genomes and, therefore, constitute a powerful tool for mapping and marker-assisted breeding. These markers are replacing microsatellites in many model and non-model plants for saturating genetic maps [10,33]. In genomes that have been poorly studied, sequence availability is the limiting factor for the discovery of SNPs.

Expressed sequenced tags (ESTs) represent a valuable sequence resource for research and breeding as they provide comprehensive information regarding the transcriptome. ESTs have played significant roles in accelerating gene discovery, allowing large-scale expression analysis, improving genome annotation, elucidating phylogenetic relationships and facilitating breeding programs for both plants and animals by providing SSRs and SNPs markers [6,8,11,34-37].

Currently, there are more than 66 million ESTs in the NCBI public collection [38]. However, less than 1,000 EST sequences are available for *Cucurbita *spp (*C. maxima*, *C. moschata *and *C. pepo*), and approximately 500,000 for all the species in the Cucurbitaceae family, most of them of cucumber and melon, included in the ICUGI Cucurbit Genomics Database [12], as compared to more than 1.5 and 2 million ESTs available for *Arabidopsis *and maize, respectively.

Recent advances in next-generation sequencing technologies allow us the large scale generation of ESTs efficiently and cost-effectively [39,40]. There are increasing studies in which 454 technologies, combined or not with Solexa/Illumina, are used to characterize transcriptomes in cereals and legumes [41-43]. Even in model species, such as *Arabidopsis*, this deep sequencing is allowing to identify new transcripts not present in previous ESTs collections [44]. Also specific transcriptomes are being generated in species for which previous genomic resources are lacking [45-47]. The new transcripts are being used for microarrays design [48], and also for high throughput SSRs or SNPs identification. SNP detection is performed by aligning raw reads from different genotypes to a reference genome or transcriptome previously available, as in maize, cucumber and even in poliploid species such as *Brassica napus *[49-51]. *De novo *assembly of raw sequences coming from a set of genotypes, followed by pairwise comparison of the overlapping assembled reads has also successfully used in species lacking any significant genomic or transcriptomic resources [52].

In this study, we describe the generation of 49,610 *Cucurbita *unigenes *de novo *assembled from about 500.000 ESTs obtained from roots, leaves and flowers of two contrasting *C. pepo *cultivars (Zucchini and Scallop, belonging to the two *C. pepo *subspecies) using Roche/454 GS FLX Titanium massive parallel pyrosequencing technology. These unigenes are functionally annotated and represent the first *C. pepo *transcriptome. They have been also screened for SSR motifs and used to identify a large SNPs collection suited for high-throughput mapping purposes. This sequence will allow accelerating genetics and breeding of this crop. It is also an important advance for cucurbit genomics as it is the first genomic resource for this genus, allowing comparisons among members of the three most economically important cucurbit genera, *Cucumis*, *Citrullus *and *Cucurbita*.

## Results and Discussion

### EST sequencing and assembly

**Figure 1:**
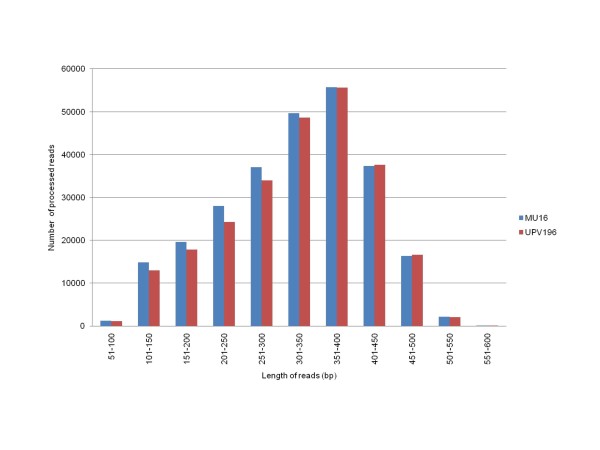
**Length distribution of the *Cucurbita *ESTs**. Data obtained after sequencing, with a half run of 454 GS FLX Titanium each one of the two *Cucurbita *cDNA libraries (Zucchini, Mu-16; Scallop, UPV-196), and processing the 454 raw reads, are presented.

We performed a half 454 GS FLX Titanium run on each of the two libraries constructed from leaves, flowers and roots from two *C. pepo *cultivars with contrasting plant, flower and fruit phenotypes, MU16 (*C. pepo *subsp. *pepo *cv Zucchini) and UPV196 (*C. pepo *subsp. *pepo *cv Scallop). A total of 407,723 and 392,370 raw sequence reads were obtained from each library (Table 1). Raw reads were processed using the Ngs_backbone software [53] to eliminate adapter sequences, low quality chromatograms and sequences of less than 100 base pairs (bp). This analysis gave rise to 261,962 and 250,789 processed sequences, comprising 164.6 Mbp of sequence, with an average length of 318.7 and 323.4 bp, respectively. The length distribution of these expressed sequence tags (ESTs) is shown in Figure 1. More than 85% ESTs fell between 200 and 500 bp in length.

**Table 1:** Sequence statistics of *Cucurbita *454 ESTs

Library	Raw reads Number/average length	Total length	Sequence quality average	Processed reads Number/average length	Total	Sequence quality average
Zucchini MU16	407,723/252	103 Mbp	31	261,962/319	84 Mbp	32

Scallop UPV196	392,370/254	100 Mbp	31	250,789/323	81 Mbp	32

TOTAL	800,093/253	203 Mbp	31	512,751/321	165 Mbp	32

Summary of the *Cucurbita pepo *expressed sequences generated with two half runs of GS-FLX Titanium pyrosequencing. Statistics of raw reads and reads after processing are indicated.

**Figure 2:**
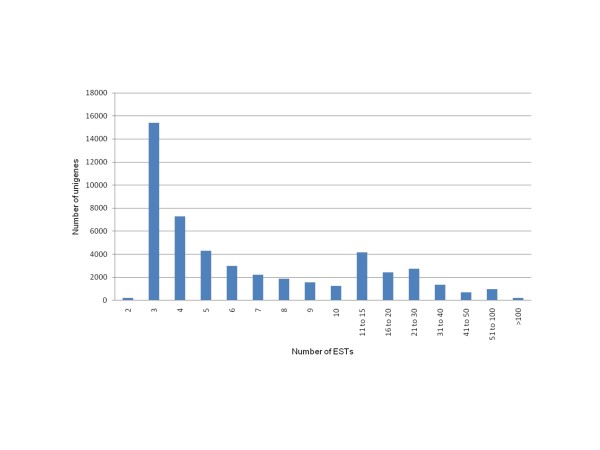
**Distribution of number of ESTs in each *Cucurbita *unigene**.

The reads produced by the GS FLX Titanium platform were used for clustering and *de novo *assembly, independently of the genotype of origin. 459,439 ESTs were finally assembled using the Mira assembler [54] yielding a total of 49,610 high-confident tentative consensus sequences (non-redundant sequences or unigenes). The distribution of the number of ESTs per unigene is shown in Figure 2. The majority of unigenes were assembled from a moderate number of ESTs (from 2 to 10), with an average of 9.3 ESTs per unigene. Of all unigenes, about a 10% contained more than 20 reads, and only 2.4% more than 50, which represented the most abundant transcripts in these cDNA libraries. This low redundancy is probably due to the success of the normalization process, responsible for the suppression of superabundant transcripts. Normalization precludes *in silico *analysis of gene expression, but greatly increases the number of unigenes that can be determined by reducing redundancy.

**Figure 3:**
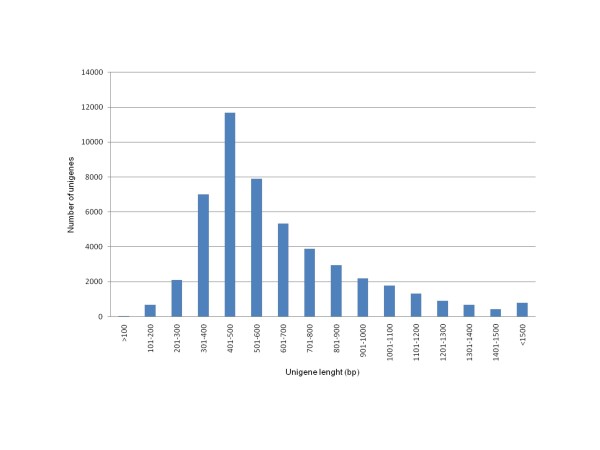
**Length distribution of the *Cucurbita *unigenes *de novo *assembled from 454 ESTs**.

The assembled unigenes had an average length of 626 bp, comprising approximately 31 Mbp in total. The length distribution of the unigenes is shown in Figure 3. The analysis revealed that more of the 50% of unigenes were larger than 537 bp, and only a 5% of the sequences were shorter than 290 bp. The number of assembled unigenes is similar to that obtained in previous transcriptome analyses in maize, *Eucaliptus*, *Artemisia*, chesnut, olive and cucumber. However, the *de novo *assembly with the longer reads obtained with the GS FLX Titanium platform render unigenes that average almost two times longer than that reported in these studies performed using 454 GS-20 and GS-FLX platforms [45-47,51,52,55]. Our assembled unigenes were also larger than that reported for American ginseng and *Glycyrrhiza uralensis *transcriptomes obtained using also the 454 GS FLX Titanium platform [56,57]. *Cucurbita *unigenes length is comparable to that reported in melon transcriptome using the conventional (Sanger) dideoxy-based sequencing [8]. These differences in length might be due to the different assemblers used. The longer unigenes present the advantage of being more accurately annotated. The raw data files are available in the Sequence Read Archive at the National Center for Biotechnology Information (NCBI) [58], accession number SRA029105.1. The sequences of the unigenes in fasta format are available in the additional file 1:'*Cucurbita *unigenes', with unigene numbers from CUTC000001 to CUTC049610.

### Structural and functional annotation

Most unigenes, 47,626 (96%) were predicted to have one ORF. By aligning the unigenes with the melon genomic sequence (available for the partners of the MELONOMICS project [7]), introns were identified in 16,697 unigenes (33.7%). Annotation results regarding ORFs and introns position are included in additional file 2: 'Annotation of ORFs and Introns'.

Codon usage was estimated using a subset of the unigenes predicted to contain full-length ORFs, with start and stop codons and without frame-shift errors. All codons were found in the dataset, with the least frequent codon represented 590 times (data not shown). As expected, the codon usage of *Cucurbita *shared some similarities with that of melon, *Arabidopsis *and other dicots. For example, T is the preferred base in the third codon position for most amino acids except for glycine, phenylalanine, serine and arginine. The preferred stop codons were UAA and UGA that occurred in the 41.4 and 41.1% of the sequences, respectively. Suppression of the CG dinucleotide in the last two codon positions is very frequent in dicots, possibly as a consequence of methylation of C in the CG dinucleotide, resulting in an increased mutation rate; the ratio XCG/XCC for *Cucurbita *was 0.69, then the suppression was more intense than in *Arabidopsis *(0.92), but milder than that reported for melon (0.52) tomato (0.58), pea (0.51), potato (0.48) or *Populus *(0.38). The GC content in third base position was similar in *Cucurbita *as compared to melon and *Arabidopsis *(46% *vs *39,9% and 42%) [8,35].

In order to identify *C. pepo *unigenes potentially encoding proteins with known function, a BLAST analysis [59] was performed in a sequential way using Swiss-Prot [60], *Arabidopsis *proteins [61], and Uniref90 [62] protein databases. Over 63% of the unigenes (31,159) had at least one significant hit (E-value cutoff = 1e-20). Most were annotated with the accurate databases Swiss-Prot (55%) and Arabidopsis (36%) and less with Uniref (9%). The majority of the unigenes have significant hits with *Arabidopsis *proteins (67%). Hits with *Cucumis *and *Cucurbita *previously reported proteins were also found.

**Figure 4:**
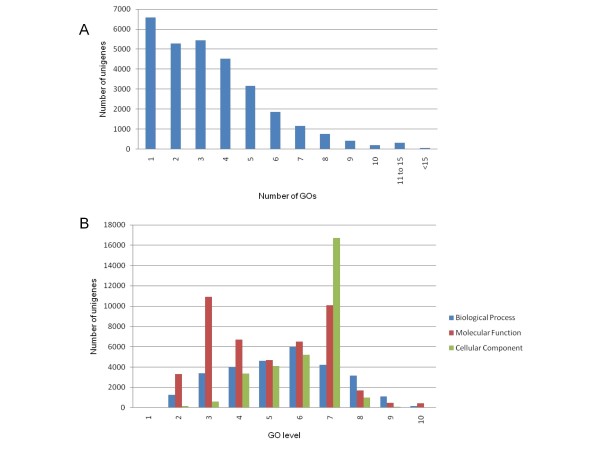
**Number of GO terms (A) and GO level distribution (B) in the annotated Cucurbita unigenes**. **A.** Distribution of GO terms in the annotated *Cucurbita *unigenes. **B.** GO level distribution in each category for the annotated *Cucurbita *unigenes.

Gene Ontology provides a structured and controlled vocabulary to describe gene products according to three categories: molecular function, biological process and cellular component [63,64]. We added GO terms using Blast2GO [65], based on the automated annotation of each unigene using BLAST results against the GenBank non redundant protein database (nr) from NCBI [66]. A total of 29,676 unigenes (60%) could be assigned to one or more ontologies. Figure 4A show the unigenes distribution regarding the number of GOs to which they were assigned. The number of GO terms per unigene varied from 1 to 34. More than the 78% of the unigenes could be assigned to more than one GO term, being the majority of the unigenes mapped to 2 to 7 GO terms. In total, 103,045 GO terms were retrieved, 25%, 47% and 28% in the biological process, in the molecular function and in the cellular component category, respectively. The distribution of annotated unigenes under different GO levels of each category (Figure 4B) shows a concentration in 4-7, 3-7 and 4-7 levels respectively for biological process, molecular function, and cellular component, indicating a good accuracy of annotation. The GO annotation analysis reinforces the idea that a broad diversity of genes was sampled in our multi-tissue normalized cDNA libraries.

**Figure 5:**
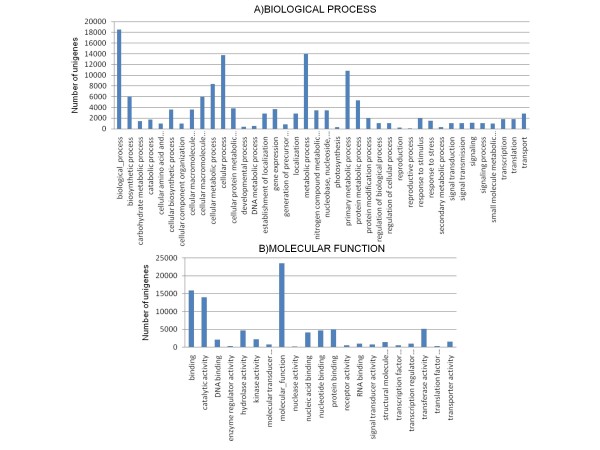
**Number of *Cucurbita *unigenes in each functional category**. *Cucurbita *unigenes were classified into different functional groups based on a set of GO slims in the A) Biological Process category and B) Molecular Function category.

We used the GO annotations to assign each unigene to a set of GO Slims of the biological process and molecular function categories, which are a list of high-level GO terms providing a broad overview of the ontology content. A summary with the number of unigenes annotated in each GO slim term is shown in Figure 5. GO annotations for the unigenes showed fairly consistent sampling of functional classes. Cellular process, metabolic process, and biosynthetic process were among the most highly represented groups under the Biological Process category. This might be indicating the analyzed tissues were undergoing rapid growth and extensive metabolic activities. Genes involved in other important biological processes such as reproduction, stress and stimulus response, signaling, and developmental processes were also identified (Figure 5A). Under the molecular function category, assignments were mainly to the catalitic and binding activities. A large number of hydrolases, kinases and transferases were annotated which suggests that this study may allow for the identification of genes involved in the secondary metabolite synthesis pathways. Also, transcription and translation factors were well represented (Figure 5B). The distribution of *Cucurbita *unigenes follow similar tendencies to that reported for *Arabidopsis *and also for the melon transcriptome [8,52,61], suggesting that the *Cucurbita *dataset could be representative of the whole squash transcriptome. All annotation results, regarding BLAST hits and GO annotations for the whole *Cucurbita *unigene collection are compiled in the additional file 3: 'Blast hits and GO terms'.

Doing a reciprocal blast search, we have also identified 19,312 *Cucurbita *unigenes (38.9%) with an ortholog in *Arabidopsis *[61] (11,022 (22.2%)) and a melon ortholog (12,461 (25.1%)) of the ICUGI data base [12] (Table 2). A list of the identified orthologs is included in additional file 4: '*Arabidopsis *and melon orthologs'.

**Table 2:** Functional annotation statistics

Database	Number of unigenes with ortholog	%	Number of orthologs
*Arabidopsis *pep	11022	22,2%	14880

Melon ICUGI	12461	25,12%	12976

Databases searched were: *Arabidopsis* and melon icugi [12,61]

Only 11,580 (23%) of the unigenes did not show significant similarity to any protein in the databases and could not be annotated. Shorter sequences are less likely to align with a significant E-value. However, the average length of these non annotated unigenes was 425 bp, with 50% being longer than 413 bp. For homology searches against known genes, unigenes longer than 200 bp are widely accepted for the effective assignment of functional annotations [57]. In previous studies performed with massive sequencing techniques a similar or even higher number of unigenes did not match with previously known sequences representing newly detected transcripts [44,49,55,57].

### Genes potentially encoding proteins involved in pathogen resistance, flowering, fruit quality and root traits

Viral and fungal pathogens affect severely the productivity of cucurbits crops. The *Cucurbita *unigene collection contains genes potentially involved in disease resistance and disease response [67-69] (see additional file 5: ' Genes potentially encoding pathogen resistance, flowering, fruit and root traits'). We have found at least one ortholog to *TOM1*, *TOM2A *(*Tobamovirus multiplication 1 and 2A*) and *THH1 *(*Tom Three Homolog 1*), genes encoding proteins that play essential roles on the tobamovirus replication [70], and also orthologs to the eukaryotic translation factors of the EIF4 family (*EIF4E*, *EIF(ISO)4E *and *EIF4EG*), known to mediate recessive resistance to poty- and other RNA viruses [71-73]. Regarding fungal responses, we have found orthologs to the *Arabidopsis RPH1 *gene (*resistance to Phytophthora 1*), involved in immune response to *Phytophthora brassicae *[74], and other genes required for non-race specific resistance to bacterial and fungal pathogens [75].The powdery mildew is the main fungal disease affecting *Cucurbita *cultivation [15]. An ortholog to *Arabidopsis PMR5 *(*powdery mildew resistant 5*) and to the *MLO10 *gene, belonging to the family of *Arabidopsis *homologs of the barley mildew resistance locus *mlo*, have been identified [76,77].

Cucurbits are models for sex determination studies due to its diverse floral sex types. Significant progress has been made in elucidating the mechanisms of plant sex determination by cloning several major sex-determining genes in cucurbit species [2,78,79]. Despite such advances, the whole mechanisms of sex determination are still unknown. Both *Cucurbita *sequenced genotypes are monoecious, but have large differences in the flowering time and the femaleness tendency. The *Cucurbita *unigene collection includes orthologs of *Arabidopsis *genes involved in flower development and flowering-time regulation (see additional file 5) [80-82]. Sex expression in cucurbits can be regulated by plant hormones and environmental factors. Ethylene is highly correlated with the femaleness and has been regarded as the primary sex determination factor [6]. Some genes related with the ethylene synthesis and also transcription factors and receptors involved in the ethylene perception and signaling have been found (various *EIN *(*Ethylene insensitive*) and *ETR *(*Ethylene response*) genes). It has been reported that auxin and also brassinosteroids can induce pistillate flower formation in part through its stimulation of ethylene production. The *Cucurbita *unigene collection has also different orthologs of proteins involved in auxin and brassinosteroids signaling, affecting flowering-time in *Arabidopsis *(*SAR*, *suppressor of auxin resistance*, *BZR*, *Brassinazole resistant*) [83-85]. Cucumber orthologs involved in those mechanisms have been reported to have differential expression in Gynoecious/hermaphroditic flowers in a recent study performed with massive sequencing [51]

We also identified a number of genes putatively involved in gibberellins (GA) biosynthetic and signaling pathways. These specific genes might be associated with the role of gibberellic acid in developmental regulation and plant stress response. The cucurbits represent a model plant system on which to examine the contents of the phloem translocation stream. A recent study reported a large-scale analysis of proteins from pumpkin (*C. maxima*) phloem exudates [5]. Identified proteins mainly corresponded to enzymes that carry out pivotal roles in stress and defense pathways. Furthermore, the detection of proteins related to GA synthesis in phloem supports the theory that the phloem is the route for transport and modification of GAs. Some orthologs of the genes encoding the main phloem sap proteins are included in our *C. pepo *collection.

Fruit development and ripening are the most important processes determining the fruit quality. At present most of the molecular and genetic data available about fruit development and ripening come from *Arabidopsis *and tomato [86]. In recent years, several genes and quantitative trait loci controlling fruit quality traits have been described in cucurbits [87,88]. As for the previously described processes, orthologs to genes involved in fruit development and quality have been found in the *Cucurbita *dataset (see additional file 5) [89,90]. These include several cell wall-metabolism enzymes and genes involved in the isoprenoid biosynthetic pathway (provides intermediates for the synthesis of sterols, carotenoids and chlorophylls, and also phytohormones and terpenes involved in plant defense). *Cucurbita *species are important sources of vitamins in many developing countries due to their high carotenoids content [91]. The *Cucurbita *unigene collection includes some enzymes involved in carotenoids biosynthesis (*PSY*, *Phytoene synthase*, *PDS*, *Phytoene desaturase *and *ZDS*, *Zeta-carotene desaturase*) [92]. The root tissue also provided some root specific genes involved in root development or stress tolerance [93].

### SSRs and SNPs discovery and validation

We performed a general screen on the *Cucurbita *unigene dataset for the presence of microsatellites, analyzing its nature and frequency [94]. A search for di-, tri-, and tetra-nucleotide repeats yielded a total of 1,935 potential SSRs in 1,822 unigenes, that is approximately 4% of the unigenes contained at least one of the considered SSR motifs. This percentage agrees with previous studies using EST databases that shows that approximately 3-7% of expressed sequences contain putative SSR motifs [34,51].

The maximum and minimum lengths of the repeats were 129 and 17, and the average length was 24 nucleotides. These were mostly tri-nucleotide (71.7%), and less di- (15.3%) and tetra- (13%). The most common repeat motifs are indicated in Table 3. A similar bias towards AG, AAG and AAAG and against CG repeats has been reported in EST-SSRs of many crops, including other cucurbits like melon and cucumber. It has been proposed that this may be due to the tendency of CpG sequences to be methylated which potentially might inhibit transcription [8,51]. Genomic SSRs identified in *C. pepo *and *C. moschata *also showed the same predominant di- and tri-nucleotide motifs [24]. The complete list of SSRs and their corresponding information are provided in additional file 6: '*Cucurbita *SSRs'.

**Table 3:** Simple Sequence repeats (SSRs) statistics

di-nucleotide repeat	Number of di-SSRs	%
AG	225	76
AT	60	20
AC	11	4
Total	296	100

tri-nucleotide repeat	Number of tri-SSRs	

AAG	699	50
AGC	135	10
ATC	116	8
AGG	99	7
AAT	89	6
Other tri-nucleotide repeats		
(% ≤ 6 each one)		
AAC, ACC, ACG,CCG, ACT	249	19
Total	1387	100

Tetra-nucleotide repeat	Number of tetra-SSRs	%

AAAT	33	13
AAAG	31	12
AATG	24	10
AATC	21	8
ATCC	18	7
AAAC	17	7
ACAT	16	6
Other tetranucleotide repeats		
(% ≤ 6 each one)		
ACTC,AACC,AAGG,ACAG,AGGC,AACG,AACT,	92	37
AATT,AGCC,AGCG,AAGC,AGGG,AGAT,ACGG, AGCT,AAGT,ACCC, ACCT		
Total	252	100

The number of di-, tri- and tetra-nucleotide repeats identified in the *Cucurbita *unigene dataset is shown for the complete set of putative SSRs.

Most SSRs (55%) were located in ORFs, being a similar number in the 5' and the 3' untranslated regions (UTRs) (Table 4). An analysis of the localization of di-, tri- and tetra-repeats showed that tri-nucleotides localized preferentially in ORFs, consistently with maintenance of the ORFs coding capacity, whereas di- and tetra-nucleotides were more frequent in UTRs. It is known that the UTRs are richer in SSRs than coding regions, particularly the 5'-UTRs [34,95]. Thus, the prevalence of tri-nucleotide repeats in the *Cucurbita *dataset may account for our high proportion of ORF-SSRs. These results agree with those reported in melon, where the most frequent SSRs in ORFs were tri-nucleotide [8].

**Table 4:** Localization of SSRS with respect to putative initiation and termination codons in the *Cucurbita *unigene dataset

	**di-SSRs**		**tri-SSRs**		**tetra-SSRs**		**all-SSRs**	
	
	N°	%	N°	%	N°	%	N°	%
5'-UTR	86	29%	172	12%	102	41%	360	19%
ORF	72	24%	903	65%	89	35%	1064	55%
3'-UTR	105	36%	194	14%	30	12%	329	17%
Other	33	11%	118	9%	31	12%	182	9%
Total	296	100%	1387	100%	252	100%	1935	100%

Unigenes were checked for the presence of the start and stop codons. "Other" means imprecise localization of the SSRs with respect to putative initiation or termination codons.

We selected a set of 30 ESTs-SSRs for validation, 26 (86.7%) amplified polymorphic fragments in a set of 10 genotypes of *Cucurbita*, 9 representative of the diversity within *C. pepo *(accessions of 3 morphotypes of spp. *pepo*, including several landraces and commercial types of the zucchini type, and two morphotypes of spp. *ovifera*) and 1 *C. moschata *accession. All but one could be transferred to the related crop *C. moschata*, producing alleles unique of this species in most cases (60%). On an average we found 3.2 alleles per primer pair in *C. pepo *and *C. moschata*. Most of EST-SSRs assayed are useful to detect variability within and between the two subspecies of C. pepo. A 77% were polymorphic between the two genotypes used for sequencing, and 50% and 88% detected variation within spp. *ovifera *and within spp. *pepo*, respectively. Also 62% detected variation among the landraces and commercial lines of zucchini. Details of these validated SSRs are included in the additional file 7: 'Validated *Cucurbita *SSRs'.

SSR markers derived from EST sequences have been extensively used in constructing genetic maps of cucurbit species [9,96]. Until recently, only a few microsatellites have been available for *Cucurbita*, and transferability from other cucurbits, such as cucumber of melon, has been demonstrated to be very low [9], then the development of SSRs for this genus is highly desirable. Gong et al. [24] developed SSRs-enriched partial genomic libraries from an Austrian oil-pumpkin variety *C. pepo *subsp. *pepo *and one accession of *C. moschata*, generating a collection of 1,058 putative SSRs. They reported a 81% validation in a set of genotypes representing the cultivar groups, also indicating a high intra-genus transferability. EST-SSRs have several advantages versus genomic; they are related to genes, being functional markers that can be used as candidate genes to study their association with phenotypic variation and the flanking sequences are more likely to be conserved among close or distant species, making their use as markers for comparative mapping easier. We will use the identified EST-SSRs markers for the construction of a genetic map, using a Recombinant Inbreed population (RILs) derived from the Zucchini (MU16) × Scallop (UPV196) cross. They will be also useful for fingerprinting commercial Zucchini cultivars, breeding lines and landraces and for genetic diversity studies within the genus, mainly performed with RAPDs or AFLPs to date [20,97].

Massive sequencing of ESTs in a number of diverse genotypes has been previously used for developing large SNPs collections [49-52]. Since the ESTs generated under the present study, using the 454 technology, are from two different cultivars belonging to two subspecies, with MU16 and UPV96 representing the 51% and 49% of the EST sequences, respectively, we expected SNPs to be frequent in our collection. The SNP calling was done with the default parameters recommended by the ngs_backbone software [53]. Stringent quality criteria were used for distinguish sequence variations from sequencing errors and mutations introduced during the cDNA synthesis step. Only variations with allele and mapping quality over the established thresholds were annotated. By applying these criteria, we initially identified a total of 19,980 SNPs and 1,174 INDELs distributed in 8,147 unigenes (16.4%), averaging a total of 2.6 single variations per unigene. Different filters were applied to facilitate the management of the variants. INDELs can be filtered out with VKS (It is not an SNP). The detailed information about the identified SNPs and INDELs is included in the additional file 8:'Cucurbita SNPs and INDELs'.

Within the detected SNPs, transitions (68%) were much more common than transversions (32%) (Table 5). A similar number of A/G and C/T transitions and also similar percentages of the four transversion types (A/T, A/C, G/T, C/G) were found. A set of SNPs could be accurately located with respect to putative initiation and termination codons, being mostly located in ORFs (82%).

**Table 5:** Single nucleotide polymorphism (SNPs) statistics

SNPs	Number	SNPs	Number
Transitions		Transversions	
A<->G	6,694	A<->T	1,793
C<->T	6,902	G<->T	1,547
		C<->G	1,548
		A<->C	1,496
Total	13,596 (68%)	Total	6,384(32%)

Type and number of transition and transversions are shown for putative high quality single nucleotide polymorphism (SNPs) identified in the *Cucurbita *database.

Other filters allowed an accurate *in silico *selection of the SNPs to identify the ones more suited for mapping purposes. All located in sequences with more than 4 SNPs or INDELs per 100 bases (filtered out with HVR4) were discarded (71, 0.36%) and also those that were variable within one or both genotypes, MU16 and UPV196 (10,937, 54.7%) (filtered out with NVSM2, NVSM1 or both filters). This requirement is intended to minimize the discovery of false polymorphisms due to the alignment of paralogs, a potentially significant problem when aligning short sequence reads. Therefore, only nucleotide variants in relatively conserved or recently derived paralogs may have been incorrectly identified as SNPs. The drawback is that some true SNPs in hotspots of genetic diversity or genes under high diversifying selection may be discarded.

From the remaining 9,043 higher confident SNPs, that were monomorphic within and polymorphic between the two sequenced genotypes, we selected a set that met different criteria for facilitating validation and for their use in a Golden Gate genotyping assay [98,99], discarding those that were closer than 60 bp to another SNP or INDEL, and/or were located closer than 60 bp to an intron and/or were closer than 60 bp to the unigene edge (filtered out with CS60, I60 and CL60, respectively). Finally, 3,538 SNPs were selected that met all criteria (see those with only a dot or a CEF tag in the filter field in additional file 8). From these, 144 SNPs were identified that can be detected as CAPS as they generate allele-specific restriction targets. We selected 50 of this putative CAPS, and 39 (80%) gave amplicons polymorphic between MU16 and UPV196 after digestion with the corresponding enzyme, which is comparable to that reported in previous studies with maize and *Eucaliptus *[49,52]. Information of the validated CAPS is included in additional file 9:'*Cucurbita *SNPs validated as CAPS'. These CAPS markers are especially useful when experience or equipment for SNPs detection using other methods is not available.

All annotation results (ORFs, introns, descriptions, GO terms, *Arabidopsis *and melon orthologs, SNVs and SSRs) have been also added in additional file 10 using the GFF3 standard file format of The Sequence Ontology Resources [100]. This format for annotations results facilitates its uploading, representation and analysis.

## Conclusions

The length and amount of the ESTs obtained with the 454 GSLX-Titanium platform has facilitated *de novo *assembly of the transcriptome in *Cucurbita pepo*, species for which limited prior sequence information is available, providing unigene sequences with length comparable to that obtained with traditional Sanger methodology. The unigene sequences constituted the first genomic resource for the *Cucurbita *genus. *Cucurbita*, along to *Cucumis *and *Citrullus*, are the three most economically important genera of the Cucurbitaceae family, whose economic importance is second only to the Solanaceae. Then this resource will enhance comparative studies within the family. The transcriptome will be important for gene discovery in *Cucurbita *and for future annotations of the *Cucurbita *genome sequence. The identified genes provide candidates for resistance genes against RNA viruses, fungal or bacterial pathogens. This is also an important resource for further study of sex determination and fruit quality in *Cucurbita*. The SSRs and SNPs identified here will constitute an important resource for mapping and marker-assisted breeding in *Cucurbita pepo *and closely related crops. The Zucchini and Scallop types are used as vegetables and highly valued in international markets, but *C. pepo *and also *C. moschata*, *C. maxima *and other minor *Cucurbita *species included a number of highly variable types that are food staples and rich sources of fat and vitamins in developing countries. All these crops will also take benefit from this genomic resource.

## Methods

### Plant material

Two cDNA libraries were constructed using material from the MU16 "Zucchini" Spanish cultivar (belonging to *Cucurbita pepo *L. ssp. *pepo*) and the UPV196 "Scallop" (belonging to *C. pepo *L. ssp. *ovifera*). Seeds of both cultivars were maintained at the COMAV Genebank. These two genotypes are representative of the sub specific variation of *C. pepo*, are readily crossable and have been selected as parentals of a RILs mapping population. They have contrasting phenotypes for traits interesting in squash breeding [15]: growth habit, sex expression, fruit shape and color, parthenocarpy tendency, shelf life, response to diseases., and are molecularly distant enough for mapping purposes [20]. Seeds were germinated and grown in trays containing a mixture of peat and sand. They were properly watered and grown at day/night temperatures of 28/20°C with a 16-h photoperiod. From each variety, tissue was sampled from the second and third true leaves, and from male and female flowers in pre and post-anthesis stage. Also the whole roots of 15 days-old plants were sampled. All tissues were collected and immediately frozen in liquid nitrogen and stored at -80°C till use.

### cDNA preparation and sequencing

Total RNA was extracted from each tissue using the TRIzol^® ^Reagent (Invitrogen, USA). We combined equivalent amounts of RNA from each tissue into two pools, one per cultivar. mRNA was purified from the total RNA using the illustraTM mRNA Purification Kit (GE Healthcare, Amersham Bioscience, Buckinghamshire, UK). Double-stranded cDNA was then synthesized from the two RNA pools with the SMART cDNA Library Construction Kit (Clontech, USA). A normalization step was carried out with TRIMMER Kit (Evrogen, Moscow, Russia) in order to prevent over-representation of the most common transcripts. The PCR products of cDNA were purified using the QIAquick PCR Purification Kit (QIAGEN, Germany). Normalization quality of cDNAs libraries was checked by quantitative PCR. The cDNA length and normalization are critical factors to have a good transcriptome representation, to have SNPs along the whole gene sequence, and to have a high quality SNP prediction. Approximately 1 μg of double-stranded cDNA from each of the two normalized cDNA pools were used for sequencing on a 454 GLX Titanium platform. A half-plate sequencing run was performed for each sample (Creative Genomics [101]). All raw sequences are available in the Sequence Read Archive at the National Center for Biotechnology Information (NCBI) [58], accession number SRA 029105.1

### cDNA sequence processing and assembly

The whole sequence analysis was carried out by using the ngs_backbone pipeline [53]. The tools and analysis mentioned in the following sections were all performed by ngs_backbone, but here the third party tools, databases, and parameters used by ngs_backbone are described.

The raw 454 sequences were processed prior to the assembly. To remove the adaptors an alignment with the adaptors used during the sequencing process was done by Exonerate [102]. The low quality regions were removed by using Lucy [103]. Sequences shorter than 100 pb were discarded and not used for the assembly. The processed 454 sequences were assembled with Mira [54]. Default ngs_backbone options for this process were used.

### *Cucurbita *gene annotation

Structural and functional annotation was performed by sequence comparison with public databases. All unique assembled sequences (unigenes) were sequentially compared using blast (cutoff e value of 1e-20) with the sequences in three major public protein databases, prioritizing handmade annotation databases. The used database order was Swiss-Prot [60], *Arabidopsis *proteins [61] and UniRef90 [62]. Once a sequence had a blast hit in one of the databases, a description was build from the description of that hit. Also, a bi-directional blast search comparison was performed in order to obtain a set of putative orthologs with *Arabidopsis *[61] and melon, using the melon unigenes contained in the Cucurbits genomic database (ICUGI).

Additionally, we performed a functional classification of the unigenes following the Gene Ontology (GO) scheme [64]. Blast2GO [65] was used for this purpose. Blast2GO used the results of a blast nr search (cutoff e value of 1e-20) to infer the relevant GO terms for every sequence. ORFs were predicted in the unigenes with the aid of the ESTScan software [104]. We used the *Arabidopsis *codon usage table to perform the ORF searching. Introns were assigned by aligning the unigenes with the melon genomic sequence using the Emboss: est2genome [105].

To assess codon usage, we used a set of the *Cucurbita *unigenes predicted to contain full-length coding regions. We performed a manual inspection, to ensure that no sequences containing frame-shift errors were included in the analysis. From this dataset, containing 4,118 sequences (529,864 codons), ORFs were defined and a codon usage table was created.

### Identification of SSRs and SNPs

SSRs were annotated using the Sputnik software [94]. Sequences containing ≥ 4 di-, tri-, or tetra-nucleotide repeats were selected. A set of SSRs were validated using the sequenced genotypes (the Zucchini MU16 and the Scallop UPV96) and a set of 7 genotypes of *C. pepo *representative of the diversity within the species: representing 3 morphotypes of spp. *pepo *(3 additional zucchini, 1 vegetable marrow, 1 pumpkin, 1 Styrian pumpkin, an oil-pumpkin variety) and one additional morphotype of spp. *ovifera *(1 croockneck) and 1 accession of *C. moschata*. Primer pairs flanking each SSR locus were designed using the Primer3 program [106]. PCR reactions were performed in a final volume of 15 μL with 1× PCR buffer (100 mM Tris-HCl, 15 mM MgCl2, 500 mM KCl, pH 8.3), 200 μM dNTPs, 0.15 μM each primer and 2 μL of template (aprox. 10 ng/μL). PCR was performed as follows: denaturation at 95°C for 3 min, followed by 10 cycles of 30 s at 95°C, 30 s at 65°C (with each cycle the annealing temperature decreasing 1°C), and of 30 s at 72°C. Products were subsequently amplified for 20 cycles at 95°C for 30 s, 55°C for 30 s and 72°C for 30 s, with a final extension at 72°C for 5 min. The forward primer was designed adding an M13 tail to its 5' end. PCR products were separated using 6% polyacrylamide gels, 1× TBE buffer in a LI-COR 4300. IRD700 and IRD800-labeled amplicons were visualized by adding to PCR mixture 0.2 μM of fluorescent label (IR700 or IR800) M13 tail.

This *Cucurbita *ESTs collection has been produced using two representatives of the two *C. pepo *subspecies appropriate for SNP discovery. Ngs_backbone was also used to detect SNVs (Single nucleotide variations, SNPs and INDELs) by mapping the 454 processed reads against the unigene assembly using BWA (Burrows-Wheeler Aligner) [107]. We only kept SNVs meeting stringent quality criteria: 1) Minimum allele quality: accumulated sequence quality for every allele; 2) Minimum mapping quality. The default threshold set by ngs_backbone was set for both parameters.

Despite satisfying the quality criteria, not all the SNVs seemed equally reliable. Several filters were applied in order to maximize a successful validation and/or implementation in high throughput genotyping platforms such as Golden gate genotyping assay [98,99]. For example, a filter to differentiate SNPs from INDELs (VKS: It is not an SNP) was applied, and also a filter that dismisses SNVs in highly variable regions (HVR4: The region has more than 4 SNVs per 100 bp). Other used filter were; CS60 (SNV is closer than 60 bp to another SNV), I60 (an intron is located closer than 60 bp), CL60 (SNV is closer than 60 bp to the sequence end), NVSM1 (SNV is variable within UPV196 or not sequenced in this genotype), NVSM2 (SNV is variable within MU16 or not sequenced in this genotype).

We also detected those SNVs that can be analyzed via CAPS (searching for alele-specific restriction targets, filter CEF) and validated a subset of them. To do this PCR conditions were used as described for SSRs and fragments digested with the corresponding enzymes were detected by agarose gel electrophoresis

## Authors' contributions

CR, JC, and BP prepared the cDNA libraries for sequencing. BP and CR selected and validated the SSRs and CAPS. JB and PZ performed the bioinformatic analysis. JC participated in the annotation analyses. BP is the main coordinator of the *Cucurbita *project and participated in the conception of the study together with JB and JC. BP was primarily responsible for drafting and revising the manuscript with contributions from co-authors. FN is the director of COMAV and critically reviewed the manuscript. All authors read and approved the final manuscript.

## Supplementary Material

Additional file 1 ***Cucurbita *unigenes**. The fasta sequence of the 49,610 *Cucurbita *unigenes assembled from 454 ESTs is included. Click here for file

Additional file 2 **Annotation of ORFs and introns**. Unigene length and predicted position of ORFs and introns is indicated for the whole *Cucurbita *unigene collection. Click here for file

Additional file 3 **Blast Hits and GO terms**. Descriptions build from the blast hit obtained by a sequential blast search of 3 protein databases [60-62] and GO annotations for the whole *Cucurbita *unigene collection are compiled. Click here for file

Additional file 4 ***Arabidopsis *and melon orthologs**. Orthologs found by reciprocal search with *Arabidopsis *and melon databases [12,61] are indicated. Click here for file

Additional file 5 **Genes potentially encoding pathogen resistance, flowering, fruit and root traits**. Genes were identified in the *Cucurbita *data set by comparison with the *Arabidopsis *and melon databases [12,61]. A brief description and the corresponding *Arabidopsis *and melon *locus *are given for each unigene. Click here for file

Additional file 6 ***Cucurbita *SSRs**. The table provides the list of SSRs identified from the *Cucurbita *unigene dataset, their length, motif sequences, position in the unigene, and scores. Click here for file

Additional file 7 **Validated *Cucurbita *SSRs**. The table provides the list of *Cucurbita *SSRs experimentally validated using a collection of *C. pepo*. Primers used for validation, number of alleles, and polymorphism detected between the sequenced genotypes, within subspecies, and within the zucchini morphotypes are included. Also transference to *C. moschata *is indicated. Click here for file

Additional file 8 ***Cucurbita *SNPs and INDELs**. The file provides the list of SNPs and INDELs (SNVs) identified from the *Cucurbita *unigene dataset, their position, the nucleotide change (or I or D for insertion and deletion in INDELs), the quality of the polymorphic base, and additional information about allele number and frequency. The different filters applied for the *in silico *selection of the SNVs are also indicated: VKS (is not an SNPs, is an INDEL), HVR4 (SNV is in a region with more than 4 SNVs per 100 bp), CS60 (SNV is closer than 60 bp to another SNV), I60 (SNV is located closer than 60 bp to an intron), CL60 (SNV is closer than 60 bp to the sequence end), NVSM1 (SNV is variable within UPV196, or not sequenced in this genotype), NVSM2 (SNV is variable within MU16, or not sequenced in this genotype), CEF (SNV does not alter a restriction target and cannot be detected as a CAPS). The VCF (Variant Call Format) version 4.0 has been used for this file [108]. Click here for file

Additional file 9 ***Cucurbita *SNPs validated as CAPS**. The table provides the list of SNPs that affected restriction targets and were validated *via *CAPS, their position, location, primers used for validation and the occurrence of polymorphism between the two sequenced varieties. Click here for file

Additional file 10 **Annotation results in GFF3**. All the annotation results (ORFS, introns, descriptions, GO terms, *Arabidopsis *and melon orthologs, SNVs and SSRs) are provided also in the standard format GFF3 of The Sequence Ontology Resources [100] that facilitate annotations uploading, representation and analysis. Click here for file
